# Supplementary material for: Murine Oncostatin M Has Opposing Effects on the Proliferation of OP9 Bone Marrow Stromal Cells and NIH/3T3 Fibroblasts Signaling through the OSMR
Source: Int J Mol Sci. 2021 Oct 28;22(21):11649. doi: 10.3390/ijms222111649 (PMC8584221; doi:10.3390/ijms222111649)
Supplement: Supplementary file 1 [file ijms-22-11649-s001.zip › ijms-1403933-supplementrary.pdf]

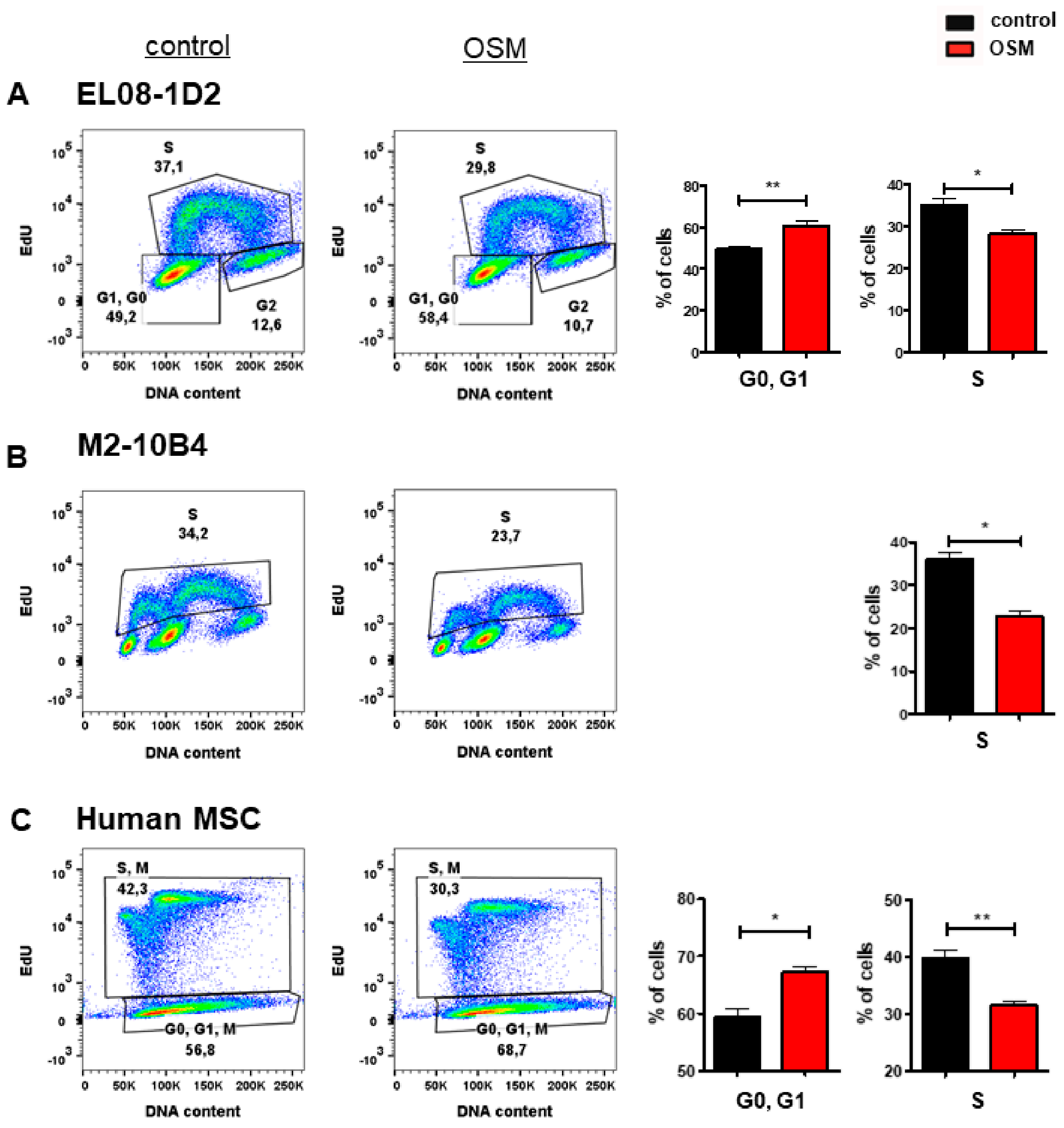

Figure S1: OSM inhibits the proliferation of murine stromal cell lines and primary human stromal cells

Representative FACS plot (left) and quantification of cell cycle phases (right) of (A) EL08-1D2, (B) M2-10B4 and (C) human primary stromal cells in presence or absence of 10 ng/mL mOSM or huOSM. Cells were cultured in medium containing 10 % FCS and treated for 24 hours. The proliferation was assessed using EdU incorporation. Cells were exposed with EdU during the last hour of treatment. The DNA content was quantified using FxCycle^{TM} Violet Stain. Student's unpaired *t*-test. *$P < 0.05$, **$P < 0.01$, and ***$P < 0.001$.

A

	OP9	OP9 + 1h OSM	OP9 + 2h OSM	OP9 + 4h OSM	OP9 + 8h OSM
LIFR	1	0.27	0.56	0.64	1.57
OSMR	1	0.09	0.42	0.25	0.19

B

	NIH/3T3	NIH/3T3 + 1h OSM	NIH/3T3 + 2h OSM	NIH/3T3 + 4h OSM	NIH/3T3 + 8h OSM
LIFR	1	0.67	1.17	1.21	0.6
OSMR	1	0.23	0.41	0.96	1.26

Figure S2: Relative quantification of immunoblots measuring LIFR and OSMR expression after mOSM treatment at different time points

(A) OP9 and (B) NIH/3T3 cells were examined for expression of LIFR and OSMR in relation to different incubation periods with 10ng/mL mOSM. Relative protein quantification was performed in comparison to the untreated control (=1).

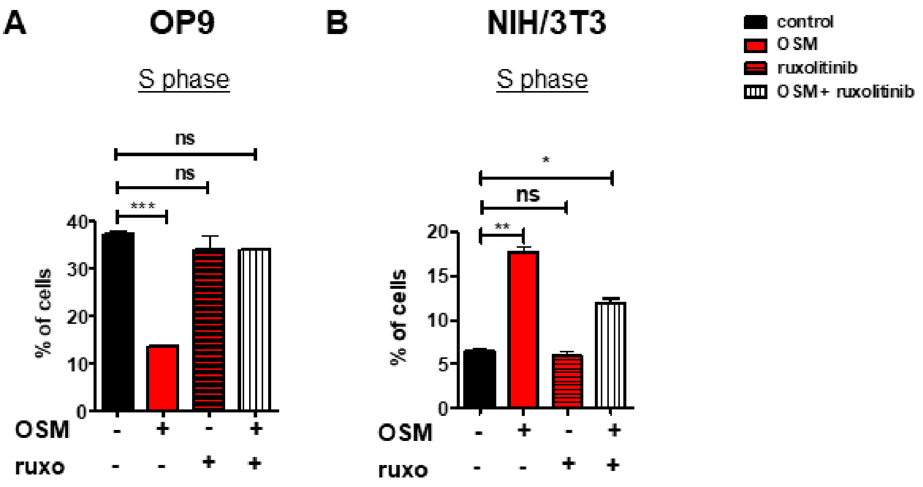

Figure S3: Ruxolitinib does not affect the proliferation of OP9 stromal cells and NIH/3T3 fibroblasts

Quantification of cell cycle phases of (A) OP9 and (B) NIH/3T3 in presence or absence of 10 ng/mL mOSM or 1 µM ruxolitinib. Cells were serum starved for 6 hours and treated for 24 hours. The proliferation was assessed using EdU incorporation. Cells were exposed with EdU during the

last 2 hours (for A) or during the last hour (for B) of treatment. The DNA content was quantified using FxCycle^{TM} Violet Stain. Student's unpaired *t*-test. *P < 0.05, **P < 0.01, and ***P < 0.001.

A

	OP9	OP9 + 15 min OSM	OP9 + 1h OSM	OP9 + 2h OSM	OP9 + 4h OSM	OP9 + 5h OSM
pRb	1	0.77	0.91	0.51	0.22	0.05

	NIH/3T3	NIH/3T3 + 15 min OSM	NIH/3T3 + 1h OSM	NIH/3T3 + 2h OSM	NIH/3T3 + 4h OSM	NIH/3T3 + 5h OSM
pRb	1	0.38	0.19	2.30	5.02	4.10

Figure S4: Relative quantification of immunoblots measuring RB activation after mOSM treatment at different time points

(A) OP9 and (B) NIH/3T3 cells were examined for expression of pRB in relation to different incubation periods with 10ng/mL mOSM. Relative protein quantification was performed in comparison to the untreated control (=1).

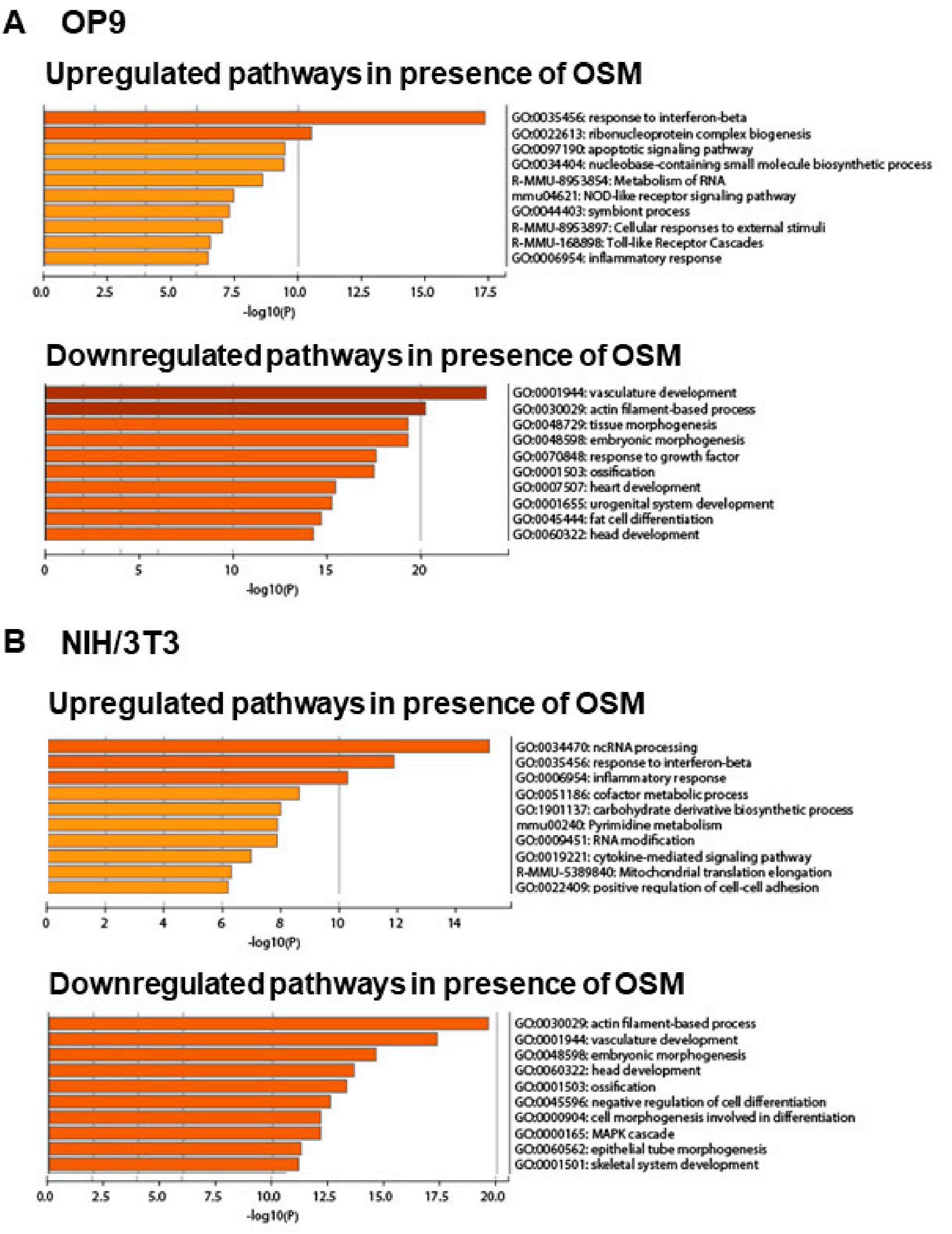

Figure S5: Pathway activation patterns were in line with the differential effects on proliferation

(A) + (B) Functional enrichment analysis by Metascape showing the top 10 enrichment clusters of up- and downregulated genes of (A) OP9 and (B) NIH/3T3 cells in presence of 10 ng/mL mOSM. Cells were starved overnight. Cells were harvested at 6 hours of mOSM treatment and RNA was isolated.

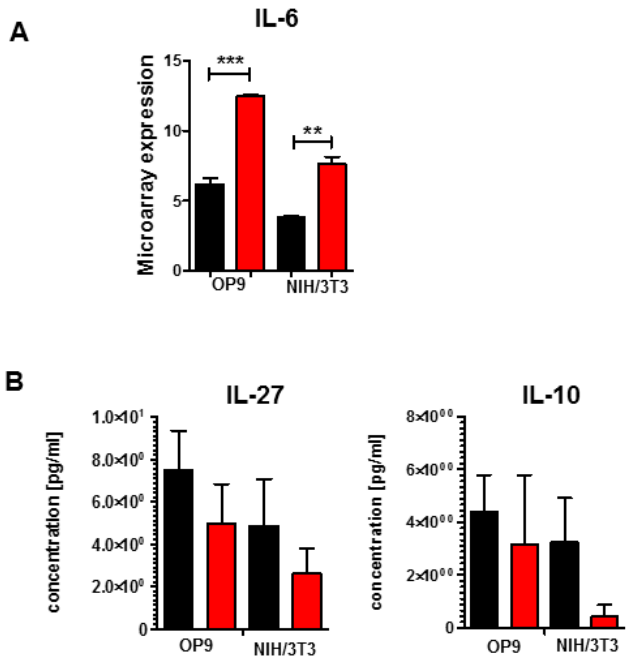

Figure S6: Cytokine secretion of OP9 and NIH/3T3 cells +/- OSM

(A) Microarray analysis of OP9 and NIH/3T3 cells +/- mOSM showing IL-6 expression. **P* < 0.05, ***P* < 0.01, and ****P* < 0.001. (B) Cytokine secretion of OP9 and NIH/3T3 cells (+/- mOSM treatment) was measured using a bead-based array detecting 13 cytokines. Student's unpaired *t*-test. **P* < 0.05, ***P* < 0.01, and ****P* < 0.001.

Table S1: shRNA sequences

shRNA	sequence
***Lifr* (shRNA 2)**	**5'TGCTGTTGACAGTGAGCGACAGGAAATTTTACAAAGATTATAGTGAAGCCACAGATGTATAATCTTTGTAAAATTTCCTGGTGCCTACTGCCTCGGA 3'**
***Lifr* (shRNA 3)**	**5'TGCTGTTGACAGTGAGCGCTCCGACTTCGTTGAAAGTGAATAGTGAAGCCACAGATGTATTCACTTTCAACGAAGTCGGATTGCCTACTGCCTCGGA 3'**
***Lifr* (shRNA 4)**	**5'TGCTGTTGACAGTGAGCGCCAGGATTGAAGGACTTACAAATAGTGAAGCCACAGATGTATTTGTAAGTCCTTCAATCCTGTTGCCTACTGCCTCGGA 3'**
***Lifr* (shRNA 5)**	**5'TGCTGTTGACAGTGAGCGCTCGTGGATGGTAGACAATAAATAGTGAAGCCACAGATGTATTTATTGTCTACCATCCACGATTGCCTACTGCCTCGGA 3'**
***Osmr* (shRNA 2)**	**5'TGCTGTTGACAGTGAGCGAACGCTTGGTTGTCTGGTTCAATAGTGAAGCCACAGATGTATTGAACCAGACAACCAAGCGTGTGCCTACTGCCTCGGA 3'**

Osmr (shRNA 3)	**5’TGCTGTTGACAGTGAGCGAAAGCTGTATCTAGATGACCTATAGTGAAGCCACAGATGTATAGGTCATCTAGATACAGCTTGTGCCTACTGCCTCGGA 3’**
Osmr (shRNA 4)	**5’TGCTGTTGACAGTGAGCGCTAGAGGAATGACTATATGTAATAGTGAAGCCACAGATGTATTACATATAGTCATTCCTCTATTGCCTACTGCCTCGGA 3’**
